# Knockout of C1q/tumor necrosis factor-related protein-9 aggravates cardiac fibrosis in diabetic mice by regulating YAP-mediated autophagy

**DOI:** 10.3389/fphar.2024.1407883

**Published:** 2024-07-08

**Authors:** Shiyan Ruan, Jun Li, Shengyun Lei, Shaomeng Zhang, Dan Xu, Anju Zuo, Linxi Li, Yuan Guo

**Affiliations:** Department of General Practice, Qilu Hospital of Shandong University, Jinan, Shandong, China

**Keywords:** diabetic cardiomyopathy, CTRP9, fibrosis, autophagy, YAP, fibroblasts

## Abstract

**Introduction:**

Diabetic cardiomyopathy (DCM) is predominantly distinguished by impairment in ventricular function and myocardial fibrosis. Previous studies revealed the cardioprotective properties of C1q/tumor necrosis factor-related protein 9 (CTRP9). However, whether CTRP9 affects diabetic myocardial fibrosis and its underlying mechanisms remains unclear.

**Methods:**

We developed a type 1 diabetes (T1DM) model in CTRP9-KO mice via streptozotocin (STZ) induction to examine cardiac function, histopathology, fibrosis extent, Yes-associated protein (YAP) expression, and the expression of markers for autophagy such LC3-II and p62. Additionally, we analyzed the direct impact of CTRP9 on high glucose (HG)-induced transdifferentiation, autophagic activity, and YAP protein levels in cardiac fibroblasts.

**Results:**

In diabetic mice, CTRP9 expression was decreased in the heart. The absence of CTRP9 aggravated cardiac dysfunction and fibrosis in mice with diabetes, alongside increased YAP expression and impaired autophagy. *In vitro*, HG induced the activation of myocardial fibroblasts, which demonstrated elevated cell proliferation, collagen production, and α-smooth muscle actin (α-SMA) expression. CTRP9 countered these adverse effects by restoring autophagy and reducing YAP protein levels in cardiac fibroblasts. Notably, the protective effects of CTRP9 were negated by the inhibition of autophagy with chloroquine (CQ) or by YAP overexpression through plasmid intervention. Notably, the protective effect of CTRP9 was negated by inhibition of autophagy caused by chloroquine (CQ) or plasmid intervention with YAP overexpression.

**Discussion:**

Our findings suggest that CTRP9 can enhance cardiac function and mitigate cardiac remodeling in DCM through the regulation of YAP-mediated autophagy. CTRP9 holds promise as a potential candidate for pharmacotherapy in managing diabetic cardiac fibrosis.

## 1 Introduction

DCM is a prevalent diabetic macrovascular complication, marked primarily by diffuse myocardial fibrosis and impairment in cardiac function (Luo et al., 2022). Fibroblasts, the most populous nonmyocyte cell type within the heart, are central to the pathophysiology of DCM (Pesce et al., 2023). When subjected to stress, these fibroblasts become activated and transdifferentiate into myofibroblasts, as evidenced by increased alpha-smooth muscle actin (α-SMA) level and heightened secretion of collagen-rich extracellular matrix (ECM), as well as enhanced cell proliferation capacity (Tallquist, 2020; Ko et al., 2022). This process results in increased cardiac wall stiffness and consequent deterioration of cardiac function. Therefore, conducting a detailed investigation into the mechanisms driving the activation and transdifferentiation of cardiac fibroblasts is critical to deepening our understanding of DCM pathogenesis and unveiling new therapeutic targets.

Recent evidence has revealed that impaired autophagy and activated Yes-associated protein (YAP) are significant contributors to cardiac fibrosis (Miyamoto, 2019; Zhang Q. et al., 2022). Autophagy, a vital cellular process for degrading and recycling damaged organelles and macromolecules via lysosomes, is indispensable for maintaining cardiac homeostasis (Klionsky et al., 2021). In diabetic models, impaired autophagy has been observed, marked by the excessive buildup of the autophagic substrate p62 and increasing levels of LC3II (Qiao et al., 2022). Autophagy-promoting drugs exhibit therapeutic efficacy against fibrosis and ventricular dysfunction in DCM (Shen et al., 2021; Zhang L. et al., 2023). These observations imply a likely involvement of autophagy in the pathogenesis of DCM. In addition, transforming growth factor-β (TGF-β), a primary fibrosis driver, has been shown to exert its effects through YAP, a downstream effector within the Hippo pathway, reinforcing YAP’s importance in fibrosis (Zhang T. et al., 2022; Weng et al., 2023). YAP is also implicated in fibrosis in other organs; its upregulation in hepatic stellate cells is linked to sustained myofibroblasts activation and increased extracellular matrix deposition in liver fibrosis (Xiang et al., 2020; Mia and Singh, 2022). Similarly, YAP overexpression in renal mesangial cells is correlated with excessive collagen production, which contributes to renal fibrosis (Choi et al., 2023). Additionally, the results showed that YAP was closely related to autophagy. Increased YAP expression in mouse proximal tubular epithelial cells inhibited autophagy, exacerbating diabetic nephropathy (Claude-Taupin et al., 2024). Recent findings also underscore the implications of YAP activation in ventricular remodeling and cardiac dysfunction in diabetic mice, suggesting that targeting YAP-mediated autophagy could be a strategic focus for DCM treatment (Ikeda et al., 2019).

C1q/tumor necrosis factor-related protein 9 (CTRP9) is a recently discovered adipokine within the CTRP superfamily, playing a pivotal role in regulating glycolipid metabolism and providing cardioprotection (Zhao et al., 2018; Guan et al., 2022). Clinical investigations have unveiled a correlation between lower CTRP9 levels and metabolic syndrome, and insulin resistance in diabetic individuals (Hwang et al., 2014; Jia et al., 2017; Moradi et al., 2018). Additionally, research has indicated that CTRP9 alleviates myocardial fibrosis postinfarction and improves fibrotic conditions in diabetic nephropathy (Hu et al., 2020; Lee et al., 2022). Nevertheless, its potential for attenuating diabetic myocardial fibrosis is not fully understood, highlighting the need for in-depth mechanistic research to clarify its therapeutic role.

This study aimed to investigate how CTRP9 ma y suppress the transdifferentiation of cardiac fibroblasts and mitigate diabetic myocardial fibrosis. We focused on determining whether CTRP9 achieves this effect by regulating the YAP-mediated autophagy pathway, which could offer a novel approach to preventing and treating DCM. To this end, a comprehensive array of *in vivo* and *in vitro* experiments was conducted.

## 2 Methods and materials

### 2.1 Animals and protocols

CTRP9 knockout (on a C57BL/6J background) mice, were generated by Shanghai Biomodel Organism Science & Technology Development Co., Ltd. STZ (MCE, USA, 55 mg/KG) in citrate buffer was injected intraperitoneally for five consecutive days to induce type 1 diabetes mellitus (T1DM) (Meng et al., 2023). Fasting glucose values above 16.7 mM were considered to be diabetes. After the mice were anesthetized with Pentobarbital Sodium (70 mg/kg, IP), animal tissues were retained for subsequent experiments. The Ethics Committee of Qiluhospital of Shandong University reviewed (KYLL-2022(ZM)-1300) and authorized all animal procedures performed with the Guide for the Care and Use of Laboratory Animals.

### 2.2 Echocardiography

Mice were anesthetized with the inhalational anesthetic isoflurane and underwent echocardiography. The induction anesthetic dose was 2%, 0.2 mg/10 g, 400 mg/kg, and the maintenance anesthetic dose was 1.2%, 0.2 mg/10 g, 240 mg/kg. The operator is unaware of the animal grouping. The left ventricle function was evaluated using a Vevo2100 imaging system (VisualSonics, Toronto, Canada). The echocardiography parameters included left ventricular end-diastolic internal diameter (LVEDD), left ventricular end-systolic diameter (LVESD), left ventricular ejection fraction (LVEF), fraction shortening (FS), early-to-late diastolic mitral flow velocities (E/A), and the ratio of early diastolic mitral inflow to mitral annular velocity (E/e’).

### 2.3 Immunohistochemistry

Hearts were prepared as 4 μm paraffin sections for subsequent staining procedures, including hematoxylin and eosin (H&E) staining as well as Masson’s trichrome staining. Anti-Collagen I (Abcam, ab34710, 1:200), Anti-Collagen III (Abcam, ab7778, 1:200), Anti-α-SMA (HUABIO, ET1607-53, 1:5000), and Anti-YAP (ABclonal, A19134, 1:200), Anti-CTRP9 (NOVUSBIO, NBP2-46834, 1:200) were applied to the paraffin sections at 4°C. Subsequent treatment included exposure to secondary antibodies (Gene Tech, GK600505) and hematoxylin staining. Three fields of view were selected for each sample, and the mean optical density was measured and then averaged. The resulting data represents one biological replicate. The results were analyzed using Image-Pro Plus 6.0 software (Media Cybernetics Inc., USA).

### 2.4 Cell culture

Primary cardiac fibroblasts were isolated from the hearts of 3- to 5-day-old C57BL/6J mice. The neonatal mouse hearts were removed and sliced into tissue fragments in pre-cooled D-Hank’s solution. The fragments were then transferred into conical flasks containing type II collagenase (Solarbio) and incubated overnight at 4°C on a shaker. The next day, the fragmented heart tissue was digested in a 37°C water bath using an EDTA-free trypsin (Solarbio) digestion solution. The collected supernatant was inoculated into flasks for cell culture. After an incubation period of 2 h at 37°C with 5% CO_2_, the fibroblasts attached to the flasks and the complete medium was subsequently substituted.

During the experiment, only cardiac fibroblasts of one to three generations were used. Primary cardiac fibroblasts were grown in complete DMEM (Gibco). The cells were preincubated with recombinant gCTRP9 (1 μg/mL) (Lei et al., 2021) for 2 h before they were exposed to a high-glucose environment (33.3 mM). Following a 48-h incubation period, the cells were harvested for analysis. CQ (1 mM) was administered 12 h before the end of the experiment to inhibit autophagy.

### 2.5 Cell transfection

Transfection of cells with the YAP overexpression plasmid (Shandong Gene & Bio Co., Ltd.) (1,000 ng/mL) and the control plasmid was conducted using Lipofectamine™ 3000 reagent (Invitrogen) in Opti-MEM™ reduced serum medium (Gibco). Complete medium was added to replace the medium 8 hours after transfection.

To knock down CTRP9, small interfering RNA (siRNA) (shandong Gene&Bio) was transfected into CFS using Lipofectamine™3000 reagent (Invitrogen) in Opti-MEM™ reduced serum medium (Gibco). The medium was replaced with complete medium 6–8 h later, and small interference was screened by detecting the mRNA expression of CTRP9 24 h later.

### 2.6 Western blotting

Extracted protein samples were underwent separation through SDS-PAGE, followed by transfer onto PVDF membrane (Millipore). Then, the membranes underwent overnight with antibodies against Collagen I (Proteintech, 66761-Ig, 1:1000), Collagen III (Abcam, ab184993, 1:1000), α-SMA (HUABIO, ET1607-53, 1:5000), GAPDH (Proteintech, 60004-1-Ig, 1:10,000), p62 (Abcam, ab109012, 1:1000), LC3B (CST, 3868S, 1:1000), and YAP (ABclonal, A19134, 1:1000), CTRP9 (NOVUSBIO, NBP2-46834, 1:500). Subsequently, secondary antibodies (HUABIO, HA1006 and HA1001) were applied to the membranes, followed by visualization using an Amersham Imager 680.

### 2.7 Immunofluorescence staining

Cardiac fibroblasts were sequentially treated with methanol on ice and 5% BSA. Subsequently, the cells were exposed to anti-α-SMA (HUABIO, ET1607-53,1:500) or anti-LC3B (CST, 3868S, 1:200) primary antibodies and left to incubate overnight. The coverslips were subjected to secondary antibody incubation, followed by staining with DAPI. Images were captured using a fluorescence microscope (Nikon Eclipse TE2000-S) or a Zeiss confocal laser scanning microscope (LSM 710, Carl Zeiss, Germany). Three fields of view were selected for each sample, and the fluorescence intensity was measured and averaged. The results obtained represented a biological replication. The ImageJ software was used for analysis.

### 2.8 qRT-PCR

FastPure Cell/Tissue Total RNA Isolation Kit V2 (Vazyme Biotech Co., Ltd.) extracted total RNA from fibroblasts and determined its concentration, followed by HiScript II Q RT SuperMix (Vazyme Biotech Co., Ltd.) and ChamQ universal SYBR qPCR Master Mix (Vazyme Biotech Co., Ltd.) for reverse transcription and PCR quantification. The primer sequences used are as follows: Collagen I-F, CCC​TGG​TCC​CTC​TGG​AAA​TG, Collagen I-R, GGA​CCt​ttg​ccc​cCT​TCT​TCT​TT; Collagen III-F, TGA​CTG​TCC​CAC​GTA​AGC​AC, Collagen III-R, GAG​GGC​CAT​AGC​TGA​ACT​GA; α-SMA-F, TTC​GTG​ACT​ACT​GCC​GAG​C, α-SMA-R, GTC​AGG​CAG​TTC​GTA​GCT​CT; p62-F, CCT​CAG​CCC​TCT​AGG​CAT​TG, p62-R, TTC​TGG​GGT​AGT​GGG​TGT​CA; LC3B-F, AGA​GCG​ATA​CAA​GGG​GGA​GA, LC3B-R, TGC​AAG​CGC​CGT​CTG​ATT​A; ATG-7-F, CCT​TCT​GGA​GCA​GTC​AGC​AA, ATG-7-R, AGG​AGC​ATG​GGG​TTT​TCG​AG; YAP1-F, TCC​AAC​CAG​CAG​CAG​CAA​AT, YAP1-R, CCT​GTT​GTT​TCA​ACC​GCA​GTC; CTRP9-F, GTG​CCC​AAG​AGT​GCT​TTC​AC, CTRP9-R, AAC​TTC​CCC​GTC​GCT​ACA​TT; GAPDH-F, TGT​CTC​CTG​CGA​CTT​CAA​CA, GAPDH-R, GGT​GGT​CCA​GGG​TTT​CTT​ACT.

### 2.9 Cell proliferation assay

The proliferation of cardiac fibroblasts was detected using Cell-Light EdU DNA Cell Proliferation Apollo567 Kit (RiBoBio). The treated cardiac fibroblasts were incubated with 10 nm EdU (5-ethynyl-2’-deoxyuridine) for 16 h, followed by subsequent experimental steps according to the instructions. Images were captured using a Zeiss fluorescence microscope, and the percentage of EdU-positive cells was analyzed using the ImageJ software.

### 2.10 Statistical analysis

The data are presented as mean ± SEM. Shapiro-Wilk test was used to evaluate the normality of the data distribution before data analysis. Statistical analyses involved Student’s t-test for comparing two groups and one-way ANOVA for assessing differences among three or more groups. Statistical analysis was performed using GraphPad Prism version 8, with significance defined as *p* < 0.05.

## 3 Results

### 3.1 CTRP9 expression was diminished within the cardiac tissue of diabetic mice

**FIGURE 1:**
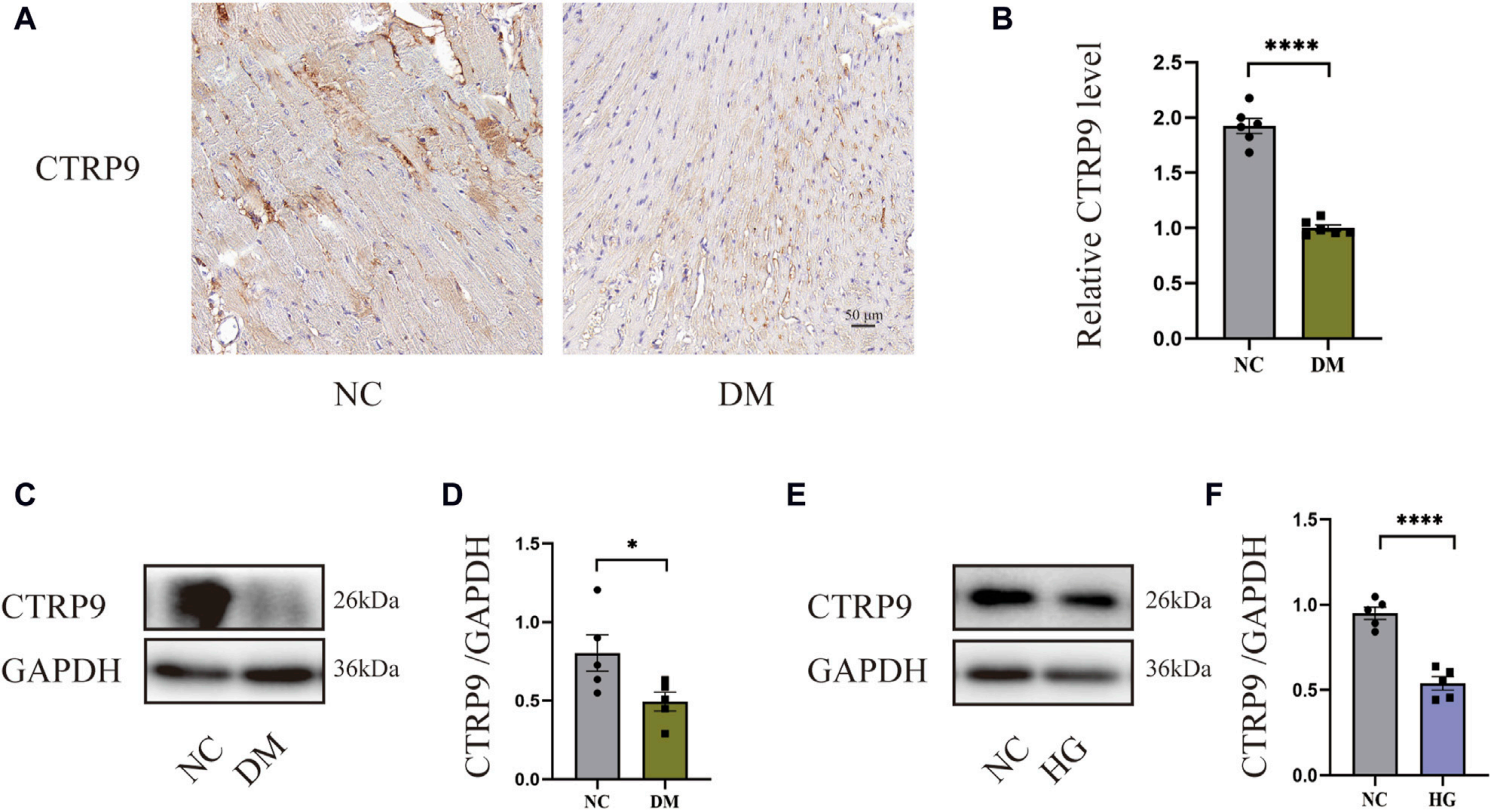
CTRP9 expression was diminished within the cardiac tissue of diabetic mice. **(A)** Illustrative immunohistochemistry images of CTRP9 in two groups. **(B)** Assessment of CTRP9 in two groups. **(C)** Illustrative Western blot of CTRP9 in animals. **(D)** Assessment of CTRP9 in two groups. **(E)** Illustrative Western blot of CTRP9 in cardiac fibroblasts. **(F)** Measurement of CTRP9 in two groups. Scale bar = 50 μm. The data are depicted as the mean ± SEM (*n* = 5–6). *p < 0.05, ****p < 0.0001.

To demonstrate the involvement of CTRP9 in cardiac fibrosis, immunohistochemistry and Western blot analysis were conducted. The findings demonstrated a marked decrease in CTRP9 expression within the hearts of diabetic mice (Figures 1A–D). *In vitro*, CTRP9 expression was identified by Western blot following the stimulation of primary cardiac fibroblasts with high glucose (HG). Fibroblast CTRP9 expression was significantly lower in the HG group compared with the group without added high glucose (Figure 1E, F).

### 3.2 CTRP9 knockout worsened cardiac dysfunction in diabetic mice

**FIGURE 2:**
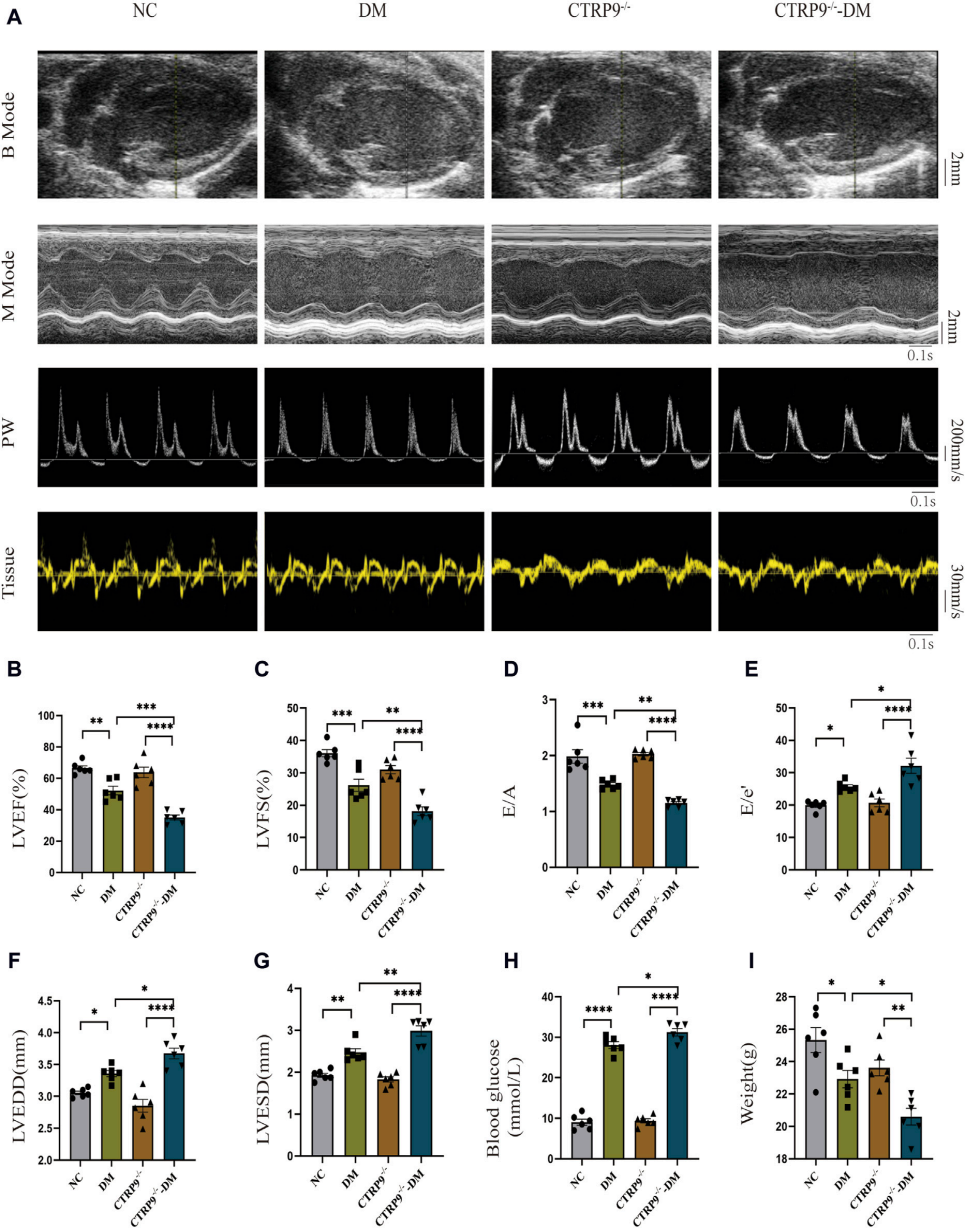
CTRP9 knockout worsened cardiac dysfunction in the mice of diabetes. **(A)** Representative B-mode (scale bar in mm), M-mode (scale bar in mm and time stamp in seconds), PW (scale bar in mm/s and time stamp in seconds) and Tissue images (scale bar in mm/s and time stamp in seconds) in four groups. **(B)** Assessment of LVEF in four groups. **(C)** Assessment of LVFS in four groups. **(D)** Assessment of E/A in four groups. **(E)** Assessment of E/e' in four groups. **(F)** Assessment of LVEDD in four groups. **(G)** Assessment of LVESD in four groups. **(H)** Blood glucose in four groups. **(I)** Body weights in four groups. The data are depicted as the mean ± SEM (*n* = 6). *p < 0.05, **p < 0.01, ***p < 0.001, ****p < 0.0001.

To elucidate the influence of CTRP9 deficiency on cardiac function, we performed echocardiograms prior to anesthesia (Figure 2A). In contrast to the NC group, the DM group demonstrated marked impairment in cardiac function, with a notable reduction in LVEF, LVFS, and E/A. These impairments were further accompanied by elevated E/e’, increased LVEDD and LVESD (Figure 2B–G). Notably, CTRP9 knockout diabetic mice exhibited greater deterioration in LVEF, LVFS, and E/A. Additionally, they exhibited increased E/e', LVEDD, and LVESD values (Figure 2B–G). Furthermore, diabetic mice in the CTRP9 deficiency group displayed significantly higher blood glucose levels and decreased body weights than those in the DM group (Figure 2H, I). In addition, in the absence of diabetes, there were no notable disparities observed in cardiac function, body weight, or blood glucose levels between the CTRP9 knockout mice and the control mice. These findings validated the successful establishment of the T1DM mouse model and demonstrated that CTRP9 deletion aggravated cardiac dysfunction in diabetic mice.

### 3.3 CTRP9 knockout exacerbated cardiac fibrosis in diabetic mice

**FIGURE 3:**
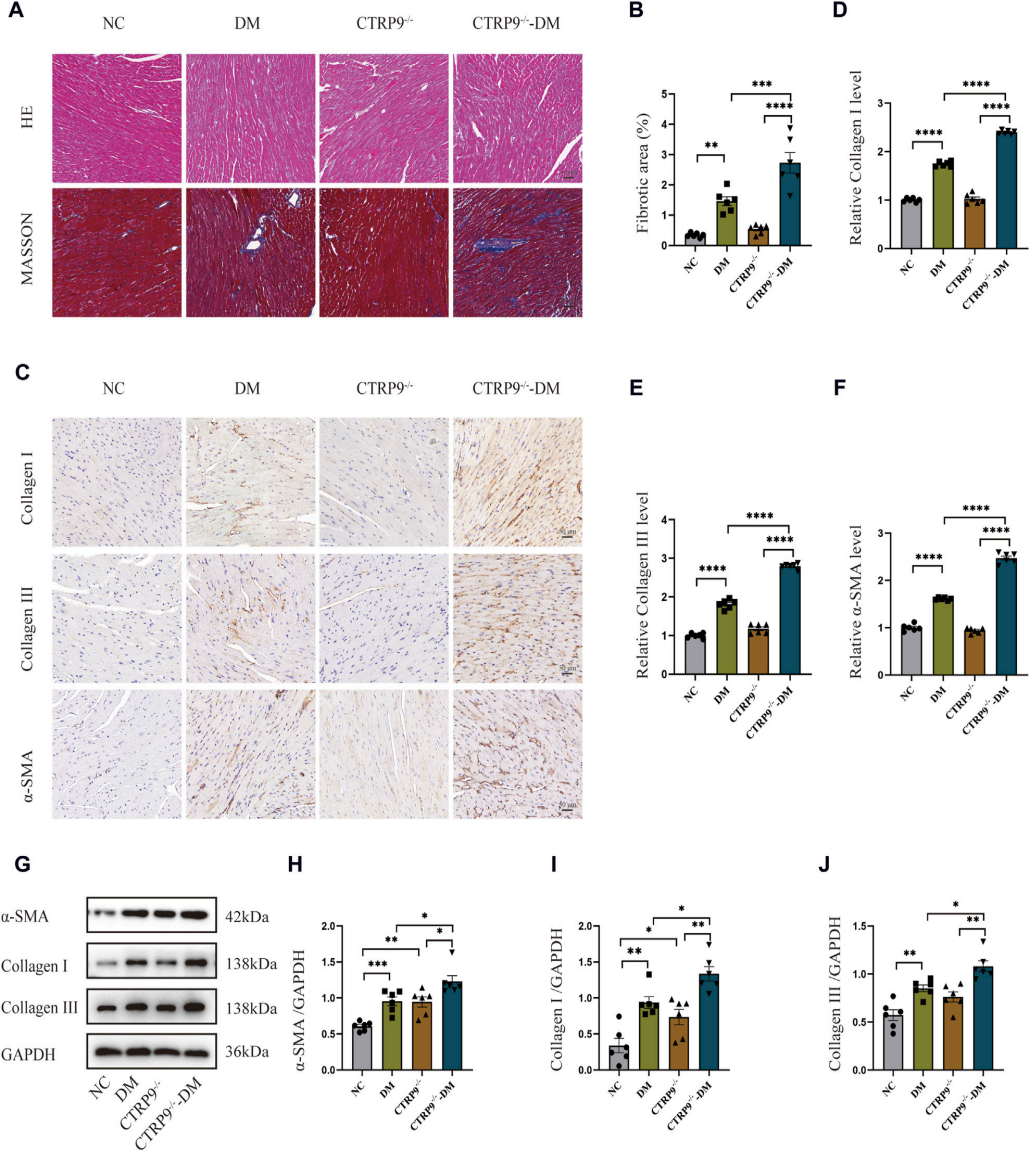
CTRP9 deletion worsened cardiac fibrosis. **(A)** Representative images of hematoxylin and eosin (H&E) and Masson’s trichrome staining in four groups. Scale bar, 50 μm. **(B)** Evaluation of the fibrotic area within four groups. **(C)** Illustrative immunohistochemistry images of Collagen I, Collagen III, and α-SMA in four groups. **(D)** Evaluation of Collagen I in four group. **(E)** Measurement of Collagen III in four group. **(F)** Relative α-SMA level in four group. **(G)** Representative blot images of α-SMA, Collagen I and Collagen III within animals. **(H)** Assessment of α-SMA in four groups. **(I)** Assessment of Collagen I within four groups. **(J)** Assessment of Collagen III in four groups. The data are depicted as the mean ± SEM (*n* = 6). *p < 0.05, **p < 0.01, ***p < 0.001, ****p < 0.0001.

To elucidate the impact of CTRP9 knockout on fibrosis within the myocardium of diabetic mice, we performed pathological staining and immunohistochemistry. Hematoxylin and eosin (H&E) staining showed significant myocardial disarray within the DM group relative to the NC group. The disruption was more severe in CTRP9 knockout mice (Figure 3A). Moreover, Masson’s trichrome staining further displayed augmented collagen accumulation within the DM group, which intensified with CTRP9 gene deletion (Figure 3A, B). Immunohistochemical analysis revealed that collagen I, collagen III, and α-SMA was significantly elevated within the DM group. This increase was even more pronounced in the absence of CTRP9, indicating a heightened fibrotic response (Figure 3C–F). Western blot analysis provided additional support for these findings, showing similar trends in protein expression (Figure 3G–J). Collectively, these results suggested that CTRP9 deletion exacerbates myocardial fibrosis in diabetic mice.

### 3.4 CTRP9 knockout aggravated autophagy inhibition and upregulated YAP expression in diabetic mice

**FIGURE 4:**
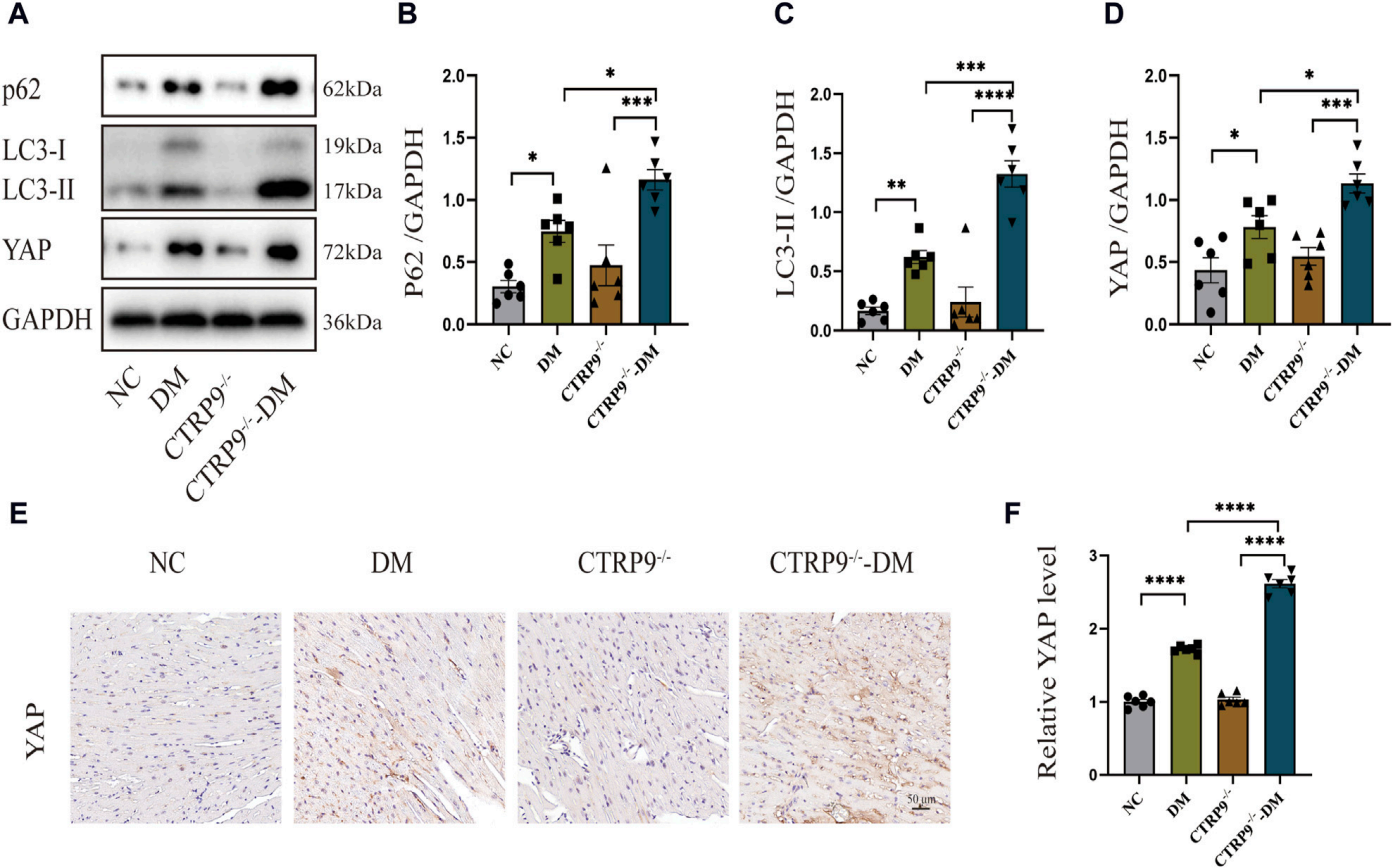
CTRP9 knockout aggravated autophagy inhibition and upregulated YAP expression in diabetic heart. **(A)** Illustrative blot images of p62, LC3-II and YAP within four groups. **(B)** Assessment of p62 in four groups. **(C)** Assessment of LC3-II in four groups. **(D)** Assessment of YAP in four groups. **(E)** Illustrative immunohistochemistry images of YAP in four groups. **(F)** Assessment of YAP in four groups. Scale bar, 50 μm. The data are depicted as the mean ± SEM (*n* = 6). *p < 0.05, **p < 0.01, ***p < 0.001, ****p < 0.0001.

Previous studies have shown a strong link between suppressed autophagy, activated YAP and the advancement of myocardial fibrosis in DCM (Ikeda et al., 2019; Wang et al., 2022). Building upon this foundation, our study examined the expression levels of p62 and LC3-II, alongside YAP, recognized for its critical involvement in organ fibrosis. Western blot analysis of cardiac tissues demonstrated a dramatic increase of p62 and LC3-II with the DM group relative to the NC group, with CTRP9 deletion exacerbating this trend further (Figure 4A–C). In parallel, YAP protein expression exhibited a significant upregulation in the DM group in comparison to the NC group, a disparity that was further amplified by CTRP9 deletion (Figure 4A, D). Immunohistochemical analysis confirmed the upregulation of YAP in the DM group, especially in the absence of CTRP9 (Figure 4E, F). These findings demonstrated that CTRP9 knockout exacerbated autophagy inhibition and upregulated YAP protein expression in diabetic mice.

### 3.5 CTRP9 treatment inhibited HG-induced myofibroblast activation

**FIGURE 5:**
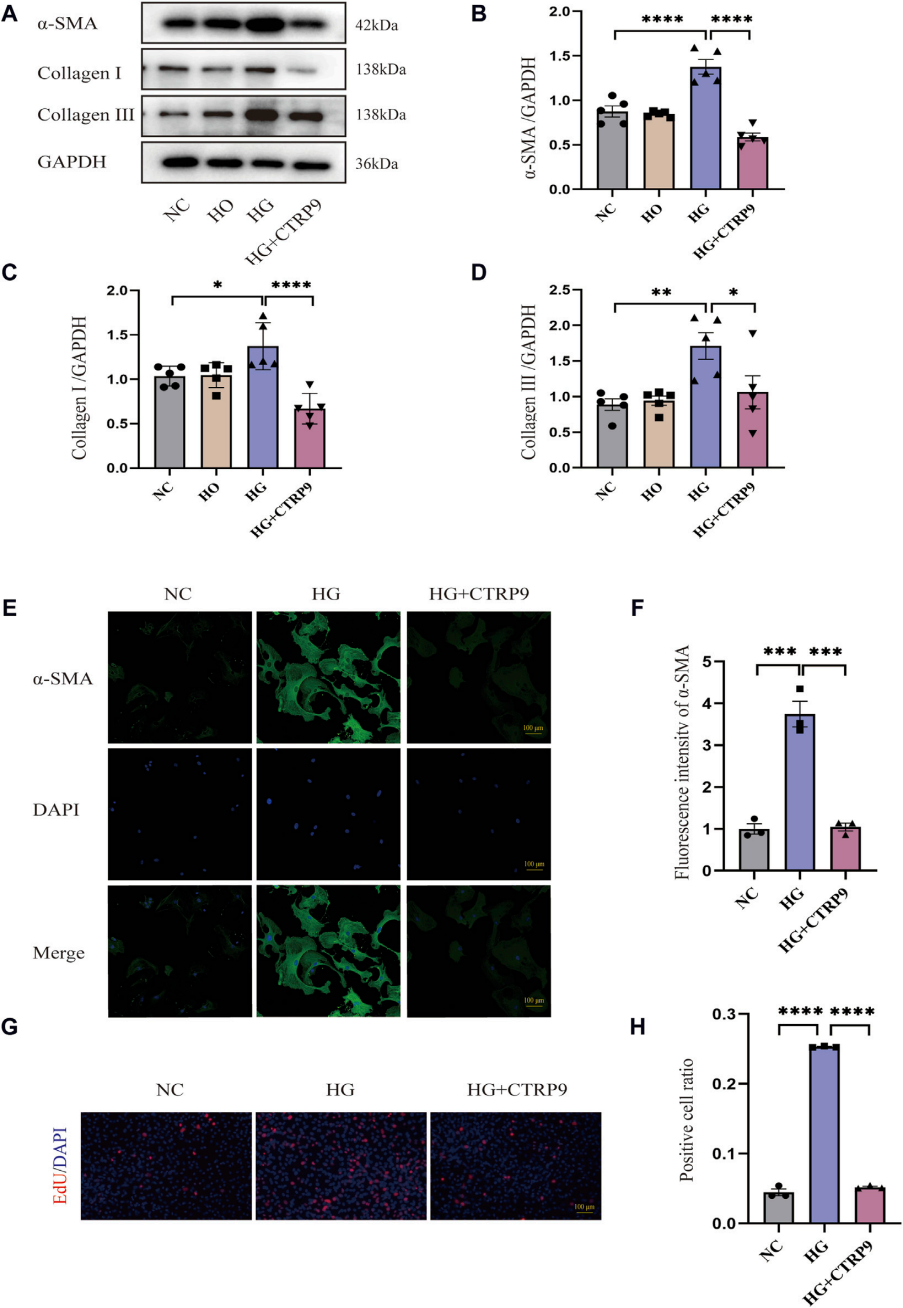
CTRP9 treatment inhibited HG-induced cardiac fibroblast activation. **(A)** Illustrative blot images of α-SMA, Collagen I and Collagen III within four groups. **(B)** Assessment of α-SMA in four groups. **(C)** Assessment of Collagen I within four groups. **(D)** Assessment of Collagen III in four groups. **(E)** Illustrative immunofluorescence images of α-SMA in three groups. **(F)**. Assessment fluorescence intensity of α-SMA in three groups. **(G)** Representative images of EdU detecting cell proliferation in three groups. **(H)** Quantification of the proportion of EdU positive cells. Scale bar, 100 μm. The data are depicted as the mean ± SEM (*n* = 3–5). *p < 0.05, **p < 0.01, ***p < 0.001, ****p < 0.0001.

Examining the influence of CTRP9 on fibroblasts activation triggered by HG, we pretreated primary cardiac fibroblasts isolated from mice with exogenous CTRP9, followed by stimulation with a 33.3 mM HG solution. Western blot analysis revealed that after 48 h of HG stimulation, myofibroblast marker α-SMA, as well as collagen level, significantly elevated within the HG group in contrast to both the NC and HO (high osmotic control) groups, whereas treatment with CTRP9 notably reduced the expression levels of these fibrosis markers (Figure 5A–D). Similar results were obtained in the RT-PCR experiment (Supplementary Figures S1A–C). Immunofluorescence staining for α-SMA further confirmed the inhibitory effect of CTRP9 on fibroblast activation (Figure 5E, F). Meanwhile, the results of the cell proliferation assay also showed that CTRP9 treatment caused a significant reduction in the level of HG-induced fibroblast proliferation (Figure 5G, H). These results suggested that CTRP9 treatment significantly inhibited myofibroblasts activation and the extracellular matrix accumulation induced by HG.

### 3.6 CTRP9 treatment inhibited HG-induced cardiac fibroblast activation by improving autophagy inhibition

**FIGURE 6:**
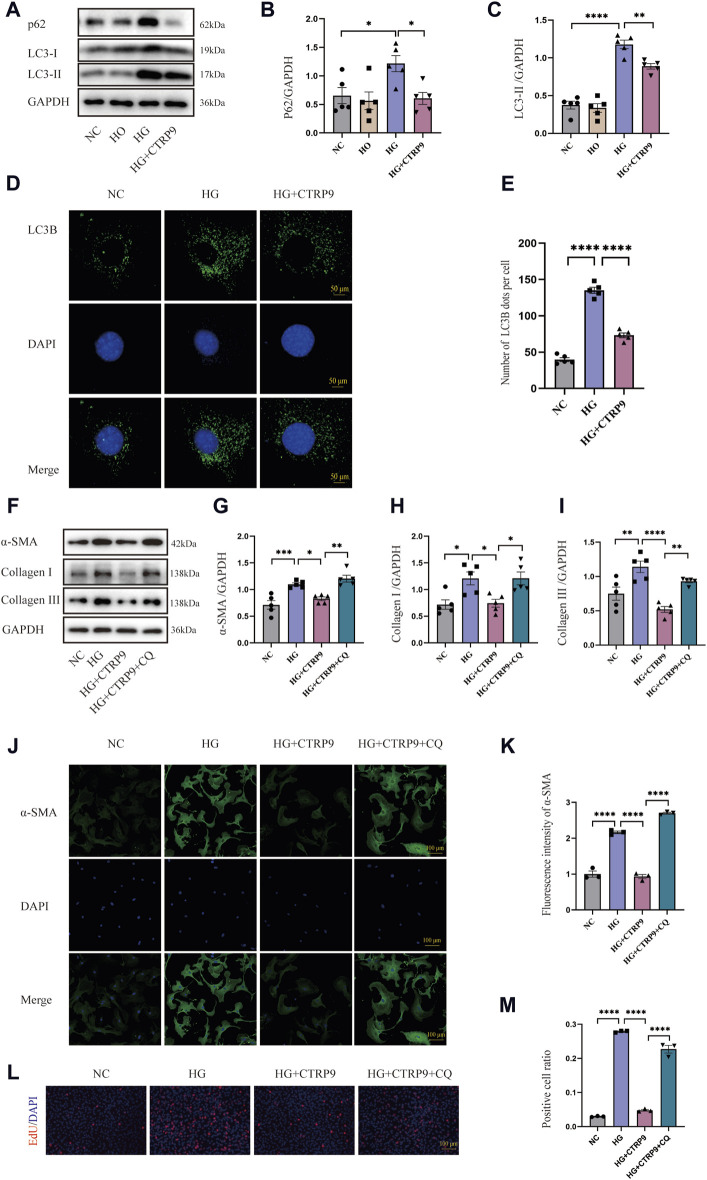
CTRP9 treatment inhibited myofibroblast activation induced by HG through improving autophagy inhibition. **(A)** Illustrative Western blot of p62 and LC3-II within four groups of cells. **(B)** Assessment of p62 in four groups. **(C)** Assessment of LC3-II in four groups. **(D)** Representative confocal microscopy images of LC3B in three groups of cells. Scale bar, 50 μm. **(E)** Quantification of LC3B puncta in three groups. **(F)** Illustrative Western blot of α-SMA, Collagen I and Collagen III within four groups of cells. **(G)** Assessment of α-SMA in four groups. **(H)** Assessment of Collagen I within four groups. **(I)** Assessment of Collagen III in four groups. **(J)** Representative immunofluorescence images of α-SMA in four groups of cells. Scale bar, 100 μm. **(K)** Quantification of fluorescence intensity of α-SMA in four groups. **(L)** Representative images of EdU detecting cell proliferation in four groups. **(M)** Quantification of the proportion of EdU positive cells. The data are depicted as the mean ± SEM (*n* = 3–5). *p < 0.05, **p < 0.01, ***p < 0.001, ****p < 0.0001.

This research sought to investigate the influence of autophagy on myofibroblast activation. Western blot analysis revealed significant upregulation of p62 and LC3-II in the HG group, suggesting possible impairment of autophagy. Notably, exogenous CTRP9 administration reduced the expression of these markers, indicating the restoration of autophagic activity (Figure 6A–C). The results of RT-PCR were consistent with the above results (Supplementary Figures S1F–G). Confocal microscopy further revealed a decrease in the number of LC3B puncta upon CTRP9 treatment, indicating a partial reversal of autophagy inhibition under HG conditions (Figure 6D, E). To investigate whether the CTRP9-mediated inhibition of cardiac fibroblast activation was autophagy dependent, cardiac fibroblasts were exposed to CQ, an autophagy inhibitor. Western blot analysis demonstrated a notable elevation of α-SMA, collagen I, and collagen III in the HG + CTRP9+CQ group compared to the HG + CTRP9 group (Figure 6F–I). The same results were obtained in the RT-PCR experiment (Supplementary Figures S2A–C). Immunofluorescence staining also revealed similar results (Figure 6J, K). In addition, the cell proliferation level in HG + CTRP9+CQ group was significantly higher than that in HG + CTRP9 group (Figure 6L, M). These combined results suggest that CTRP9 can suppress fibroblast activation and extracellular matrix secretion through restoring autophagy disrupted by high-glucose conditions.

### 3.7 CTRP9 treatment inhibited HG-induced myofibroblast activation through the YAP-mediated autophagy pathway

**FIGURE 7:**
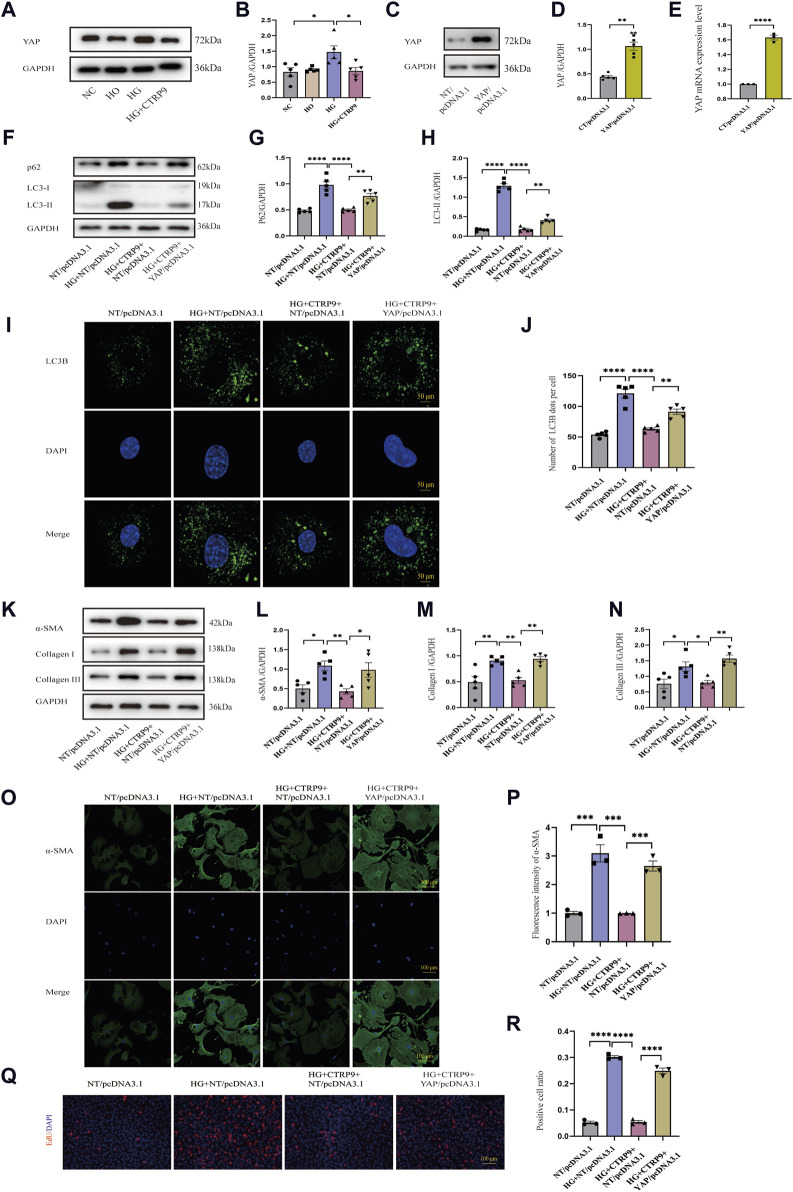
CTRP9 treatment inhibited HG-induced activation through the YAP-mediated autophagy pathway. **(A)** Illustrative Western blot of YAP in four groups of cells. **(B)** Assessment of YAP in four groups. **(C, D)** Illustrative Western blot images of YAP overexpression and their quantification in two groups. **(E)** Quantification YAP mRNA expression level in two groups. **(F)** Illustrative Western blot of p62 and LC3-II with four groups. **(G)** Assessment of p62 in four groups. **(H)** Assessment of LC3-II in four groups. **(I)** Representative confocal microscopy images of LC3B in four groups of cells. Scale bar, 50 μm. **(J)** Quantification of LC3B puncta in four groups. **(K)** Illustrative Western blot of α-SMA, Collagen I and Collagen III within four groups of cells. **(L)** Assessment of α-SMA in four groups. **(M)** Assessment of Collagen I within four groups. **(N)** Assessment of Collagen III in four groups. **(O)** Representative immunofluorescence images of α-SMA in four groups of cells. Scale bar, 100 μm. **(P)** Quantification of fluorescence intensity of α-SMA in four groups. **(Q)** Representative images of EdU detecting cell proliferation in four groups. Scale bar, 100 μm. **(R)** Quantification of the proportion of EdU positive cells. The data are presented as the mean ± SEM (*n* = 3–5). *p < 0.05, **p < 0.01, ***p < 0.001, ****P < 0.0001.

Given that YAP is closely associated with fibrosis (Francisco et al., 2020), we elucidated its role in cardiac fibroblast activation. Western blot revealed notable YAP increase within the high glucose (HG) group relative to both the NC and HO groups, and the addition of exogenous CTRP9 led to a significant suppression of YAP expression (Figure 7A, B). The mRNA change level of YAP was consistent with the protein level (Supplementary Figure S1D). To determine the impact of YAP on autophagy and its potential role in mediating the regulatory effect of CTRP9 on cardiac fibroblast activation, we overexpressed YAP in primary mouse cardiac fibroblasts via plasmid-mediated transfection (Figure 7C–E). We observed that overexpression of YAP eliminated CTRP9-mediated autophagy recovery (Figure 7F–J). The experimental results of RT-PCR also support the above results (Supplementary Figures S3E, F). Additionally, overexpressing YAP abolished the inhibitory effect exerted by CTRP9 toward myofibroblast activation. In the group overexpressing YAP, the levels of fibrosis markers and collagen were significantly elevated compared to the group treated with HG + CTRP9+NT/pcDNA3.1(Figure 7K–N). The results of RT-PCR were consistent with the above results (Supplementary Figures S3A–C). Immunofluorescence staining for α-SMA provided additional confirmation of these effects (Figure 7O–P). Overexpression of YAP significantly increased the proliferation of cardiac fibroblasts treated with high glucose and CTRP9 (Figure 7Q–R). Based on the observations, CTRP9 suppresses high glucose-induced cardiac fibroblast activation and extracellular matrix deposition through YAP-mediated autophagy.

## 4 Discussion

**FIGURE 8:**
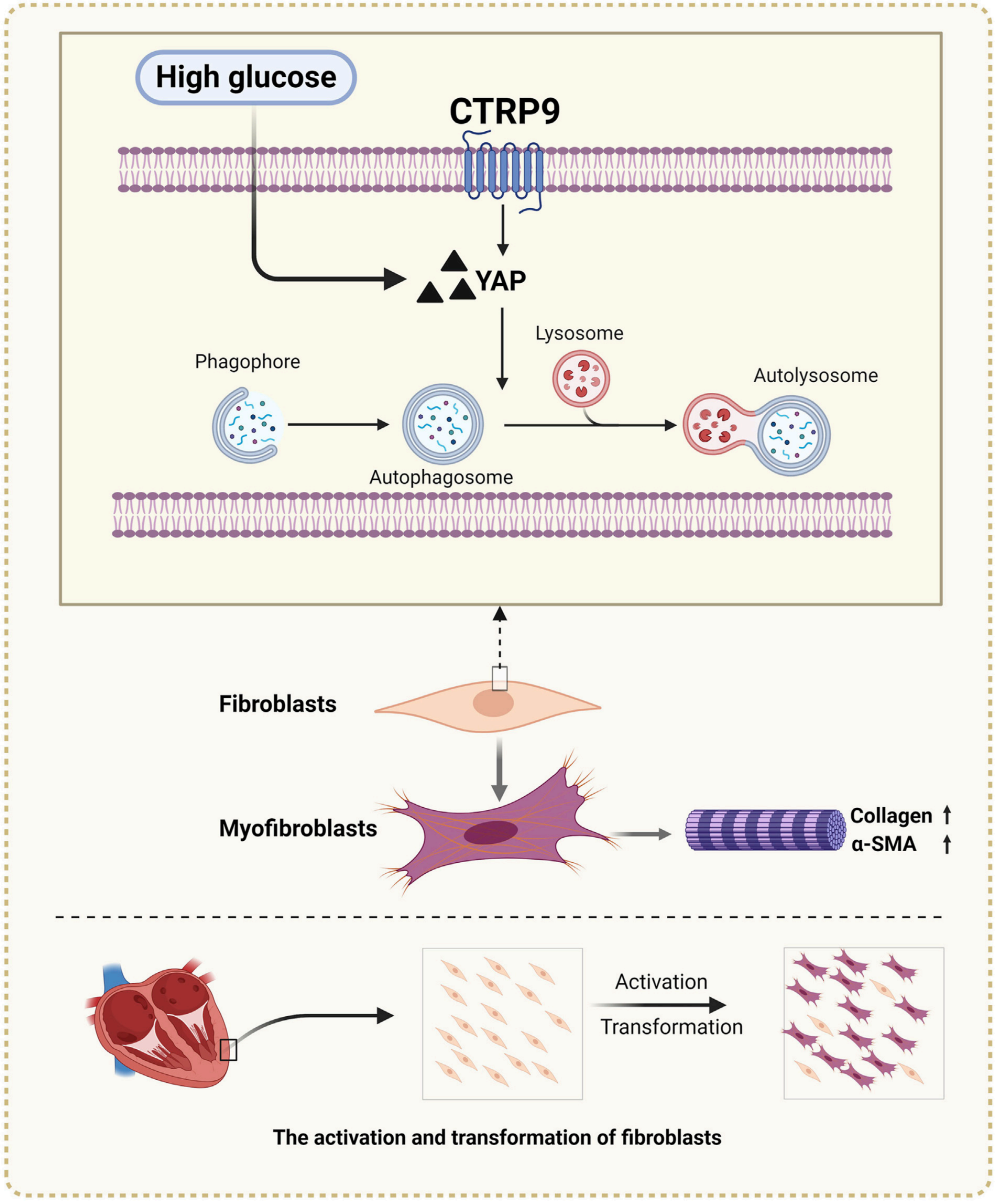
Diagram of CTRP9 attenuating cardiac fibrosis in diabetic mice though affecting YAP-mediated autophagy pathway. Created with BioRender.com.

In this study, we observed that CTRP9 knockout aggravated cardiac fibrosis and dysfunction in diabetic mice, indicating its potential protective role against cardiac complications associated with diabetes. This exacerbation was accompanied by a marked reduction in cardiac autophagy and an increase in YAP protein levels, suggesting a regulatory imbalance in diabetic conditions due to CTRP9 deficiency (Figure 8). Our *in vitro* experiments further demonstrated that supplementation with CTRP9 attenuated cardiac fibroblast activation and improved their fibrotic profile, primarily by restoring autophagy, which was impaired by HG exposure. Moreover, we observed that CTRP9 supplementation effectively suppressed the expression of YAP, a protein closely linked to the fibrotic process, whereas YAP overexpression counteracted the autophagy-restoring and antifibrotic effects of CTRP9. Together, these findings indicated that CTRP9 mitigated diabetic cardiac fibrosis through the regulation of YAP-mediated autophagy.

Autophagy, a key homeostatic pathway highly conserved in cells for degrading and recycling macromolecules and damaged organelles, serves as an important guardian of quality control in cardiac cells (Dewanjee et al., 2021). In the context of diabetes, however, autophagy often becomes dysfunctional in the heart. (Zang et al., 2020). Previous studies have demonstrated that sustained hyperglycemia impairs cardiomyocyte autophagy, resulting in elevated cardiac collagen deposition and cardiac dysfunction in diabetic mice (Xue et al., 2022). Conversely, Metrnl, by activating the AMPK pathway, has been found to restore suppressed autophagy in the heart, providing a safeguard against DCM (Lu et al., 2023). However, the exact contribution of autophagy to cardiac health in DCM is subject to debate, as contrasting evidence exists. Some findings suggest that certain diabetic hearts exhibit autophagic hyperactivation, which has been shown to have a detrimental effect on the heart (Guo et al., 2020; Jiang et al., 2022). Such disparate conclusions could arise from differences in diabetes types, stages, severity, and concurrent medical conditions across studies. In our current work, we developed a T1DM mouse model using successive low-dose intraperitoneal injections of STZ. Our observations revealed markedly reduced autophagic activity in the hearts of mice with diabetes, which was further exacerbated by CTRP9 knockout. This finding was supported by *in vitro* evidence indicating that CTRP9 pretreatment can mitigate HG-induced autophagy inhibition in cardiac fibroblasts and improve their fibrotic state. In contrast, autophagy suppression by CQ counteracted the beneficial effects of CTRP9. These findings elucidated the critical role of inhibited autophagy in diabetic myocardial fibrosis and suggested that autophagy restoration by CTRP9 represents a promising therapeutic avenue for DCM.

YAP, a pivotal constituent within the Hippo pathway, subjects to negative regulation within this pathway (Ibar and Irvine, 2020). Stimulating the Hippo pathway prompts kinases such as mammalian sterile 20-like protein kinase 1/2 (Mst1/2) and large tumor suppressor (Lats1/2) to phosphorylate YAP, causing its sequestration in the cytoplasm and subsequent degradation (Choi et al., 2024). During Hippo pathway inactivation, YAP *undergoes* dephosphorylation and translocates to the nucleus, where it activates genes that drive its biological roles (Kiang et al., 2024). Elevated YAP expression is a common feature of fibrosis in various organs, including pulmonary, hepatic, and renal fibrosis, and has been increasingly associated with cardiac pathologies (Stancil et al., 2021; Zhang J. et al., 2023; Chitturi et al., 2023). YAP activation results in myocardial hypertrophy and fibrosis (Garoffolo et al., 2022; Kashihara et al., 2022). Moreover, YAP is activated in resident cardiac fibroblasts postmyocardial infarction, leading to adverse remodeling and even heart failure (Mia et al., 2022). Our study showed that absence of the CTRP9 gene resulted in increased YAP expression within the diabetic heart. *In vitro*, the addition of CTRP9 to cardiac fibroblasts inhibited the HG-induced upregulation of YAP. To further elucidate the impact of YAP and CTRP9 on ameliorating HG-induced cardiac fibroblast activation, we overexpressed YAP with plasmids to counteract the inhibitory effect of CTRP9 on HG-triggered cardiac fibroblast transdifferentiation and collagen secretion. Furthermore, growing evidence suggests that YAP may regulate autophagy levels as an upstream mechanism. Activation of YAP can lead to autophagy inhibition (Claude-Taupin et al., 2023; Wu et al., 2024). However, there is also evidence that autophagy controls YAP expression. When autophagy is active, YAP interacts with the receptor protein p62 of the autophagy pathway and is degraded by autophagic lysosomes (Hao et al., 2024). Our *in vitro* experiments showed that overexpression of YAP counteracted the autophagy restoring effect of CTRP9.

However, our study is not without its limitations. First, we established an STZ-induced T1DM mouse model, as T1DM mice develop both diastolic and systolic dysfunction, which closely resemble the cardiac impairments observed in clinical diabetic patients. Type 2 diabetes mellitus primarily presents with diastolic dysfunction, and creating type 2 diabetic animal models necessitates more intricate environmental interventions, such as high-fat diets and a lack of physical activity, potentially leading to highly variable experimental outcomes. Second, while the current literature acknowledges the multifaceted interaction between YAP and autophagy, our research did not explore this relationship in depth. Delving into the nuanced interplay between YAP and autophagy is a primary aim of subsequent investigations.

In conclusion, we demonstrated that CTRP9 knockout exacerbated diabetic myocardial fibrosis by inhibiting autophagy and upregulating YAP expression. Our investigation provides valuable mechanistic understanding regarding the therapeutic implications of CTRP9 in diabetic myocardial fibrosis, laying a foundation for the advancement of novel CTRP9-based pharmaceutical interventions.

## Data Availability

The datasets presented in this study can be found in online repositories. The names of the repository/repositories and accession number(s) can be found in the article/Supplementary Material.

## References

[B1] Chitturi P. Xu S. Ahmed Abdi B. Nguyen J. Carter D. E. Sinha S. (2023). Tripterygium wilfordii derivative celastrol, a YAP inhibitor, has antifibrotic effects in systemic sclerosis. Ann. Rheum. Dis. 82 (9), 1191–1204. 10.1136/ard-2023-223859 37328193

[B2] Choi S. Hong S. P. Bae J. H. Suh S. H. Bae H. Kang K. P. (2023). Hyperactivation of YAP/TAZ drives alterations in mesangial cells through stabilization of N-myc in diabetic nephropathy. J. Am. Soc. Nephrol. 34 (5), 809–828. 10.1681/asn.0000000000000075 36724799 PMC10125647

[B3] Choi S. Kang J. G. Tran Y. T. H. Jeong S. H. Park K. Y. Shin H. (2024). Hippo-YAP/TAZ signalling coordinates adipose plasticity and energy balance by uncoupling leptin expression from fat mass. Nat. Metab. 6 (5), 847–860. 10.1038/s42255-024-01045-4 38811804 PMC11136666

[B4] Claude-Taupin A. Isnard P. Bagattin A. Kuperwasser N. Roccio F. Ruscica B. (2023). The AMPK-Sirtuin 1-YAP axis is regulated by fluid flow intensity and controls autophagy flux in kidney epithelial cells. Nat. Commun. 14 (1), 8056. 10.1038/s41467-023-43775-1 38052799 PMC10698145

[B5] Claude-Taupin A. Terzi F. Codogno P. Dupont N. (2024). Yapping at the autophagy door? The answer is flowing in the kidney proximal tubule. Autophagy, 1–2. 10.1080/15548627.2024.2319023 PMC11210927 38362917

[B6] Dewanjee S. Vallamkondu J. Kalra R. S. John A. Reddy P. H. Kandimalla R. (2021). Autophagy in the diabetic heart: a potential pharmacotherapeutic target in diabetic cardiomyopathy. Ageing Res. Rev. 68, 101338. 10.1016/j.arr.2021.101338 33838320

[B7] Francisco J. Zhang Y. Jeong J. I. Mizushima W. Ikeda S. Ivessa A. (2020). Blockade of fibroblast YAP attenuates cardiac fibrosis and dysfunction through MRTF-A inhibition. JACC Basic Transl. Sci. 5 (9), 931–945. 10.1016/j.jacbts.2020.07.009 33015415 PMC7524792

[B8] Garoffolo G. Casaburo M. Amadeo F. Salvi M. Bernava G. Piacentini L. (2022). Reduction of cardiac fibrosis by interference with YAP-dependent transactivation. Circ. Res. 131 (3), 239–257. 10.1161/circresaha.121.319373 35770662

[B9] Guan H. Wang Y. Li X. Xiang A. Guo F. Fan J. (2022). C1q/Tumor necrosis factor-related protein 9: basics and therapeutic potentials. Front. Physiol. 13, 816218. 10.3389/fphys.2022.816218 35370782 PMC8971810

[B10] Guo X. Lin H. Liu J. Wang D. Li D. Jiang C. (2020). 1,25-Dihydroxyvitamin D attenuates diabetic cardiac autophagy and damage by vitamin D receptor-mediated suppression of FoxO1 translocation. J. Nutr. Biochem. 80, 108380. 10.1016/j.jnutbio.2020.108380 32299030

[B11] Hao Y. Feng D. Ye H. Liao W. (2024). Nobiletin alleviated epithelial-mesenchymal transition of hepatocytes in liver fibrosis based on autophagy-hippo/YAP pathway. Mol. Nutr. Food Res. 68 (3), e2300529. 10.1002/mnfr.202300529 38044268

[B12] Hu H. Li W. Liu M. Xiong J. Li Y. Wei Y. (2020). C1q/Tumor necrosis factor-related protein-9 attenuates diabetic nephropathy and kidney fibrosis in db/db mice. DNA Cell Biol. 39 (6), 938–948. 10.1089/dna.2019.5302 32283037

[B13] Hwang Y. C. Woo Oh S. Park S. W. Park C. Y. (2014). Association of serum C1q/TNF-Related Protein-9 (CTRP9) concentration with visceral adiposity and metabolic syndrome in humans. Int. J. Obes. (Lond) 38 (9), 1207–1212. 10.1038/ijo.2013.242 24357853

[B14] Ibar C. Irvine K. D. (2020). Integration of hippo-YAP signaling with metabolism. Dev. Cell 54 (2), 256–267. 10.1016/j.devcel.2020.06.025 32693058 PMC7373816

[B15] Ikeda S. Mukai R. Mizushima W. Zhai P. Oka S. I. Nakamura M. (2019). Yes-associated protein (YAP) facilitates pressure overload-induced dysfunction in the diabetic heart. JACC Basic Transl. Sci. 4 (5), 611–622. 10.1016/j.jacbts.2019.05.006 31768477 PMC6872826

[B16] Jia Y. Luo X. Ji Y. Xie J. Jiang H. Fu M. (2017). Circulating CTRP9 levels are increased in patients with newly diagnosed type 2 diabetes and correlated with insulin resistance. Diabetes Res. Clin. Pract. 131, 116–123. 10.1016/j.diabres.2017.07.003 28743061

[B17] Jiang K. Xu Y. Wang D. Chen F. Tu Z. Qian J. (2022). Cardioprotective mechanism of SGLT2 inhibitor against myocardial infarction is through reduction of autosis. Protein Cell 13 (5), 336–359. 10.1007/s13238-020-00809-4 33417139 PMC9008115

[B18] Kashihara T. Mukai R. Oka S. I. Zhai P. Nakada Y. Yang Z. (2022). YAP mediates compensatory cardiac hypertrophy through aerobic glycolysis in response to pressure overload. J. Clin. Investig. 132 (6), e150595. 10.1172/jci150595 35133975 PMC8920343

[B19] Kiang K. M. Ahad L. Zhong X. Lu Q. R. (2024). Biomolecular condensates: hubs of Hippo-YAP/TAZ signaling in cancer. Trends Cell Biol. 10.1016/j.tcb.2024.04.009 38806345

[B20] Klionsky D. J. Petroni G. Amaravadi R. K. Baehrecke E. H. Ballabio A. Boya P. (2021). Autophagy in major human diseases. Embo J. 40 (19), e108863. 10.15252/embj.2021108863 34459017 PMC8488577

[B21] Ko T. Nomura S. Yamada S. Fujita K. Fujita T. Satoh M. (2022). Cardiac fibroblasts regulate the development of heart failure via Htra3-TGF-β-IGFBP7 axis. Nat. Commun. 13 (1), 3275. 10.1038/s41467-022-30630-y 35672400 PMC9174232

[B22] Lee S. M. Lee J. W. Kim I. Woo D. C. Pack C. G. Sung Y. H. (2022). Angiogenic adipokine C1q-TNF-related protein 9 ameliorates myocardial infarction via histone deacetylase 7-mediated MEF2 activation. Sci. Adv. 8 (48), eabq0898. 10.1126/sciadv.abq0898 36459558 PMC10936044

[B23] Lei S. Chen J. Song C. Li J. Zuo A. Xu D. (2021). CTRP9 alleviates foam cells apoptosis by enhancing cholesterol efflux. Mol. Cell Endocrinol. 522, 111138. 10.1016/j.mce.2020.111138 33352225

[B24] Lu Q. B. Ding Y. Liu Y. Wang Z. C. Wu Y. J. Niu K. M. (2023). Metrnl ameliorates diabetic cardiomyopathy via inactivation of cGAS/STING signaling dependent on LKB1/AMPK/ULK1-mediated autophagy. J. Adv. Res. 51, 161–179. 10.1016/j.jare.2022.10.014 36334887 PMC10491969

[B25] Luo W. Lin K. Hua J. Han J. Zhang Q. Chen L. (2022). Schisandrin B attenuates diabetic cardiomyopathy by targeting MyD88 and inhibiting MyD88-dependent inflammation. Adv. Sci. (Weinh) 9 (31), e2202590. 10.1002/advs.202202590 36180407 PMC9631063

[B26] Meng L. Lu Y. Wang X. Cheng C. Xue F. Xie L. (2023). NPRC deletion attenuates cardiac fibrosis in diabetic mice by activating PKA/PKG and inhibiting TGF-β1/Smad pathways. Sci. Adv. 9 (31), eadd4222. 10.1126/sciadv.add4222 37531438 PMC10396312

[B27] Mia M. M. Cibi D. M. Ghani S. Singh A. Tee N. Sivakumar V. (2022). Loss of Yap/Taz in cardiac fibroblasts attenuates adverse remodelling and improves cardiac function. Cardiovasc Res. 118 (7), 1785–1804. 10.1093/cvr/cvab205 34132780

[B28] Mia M. M. Singh M. K. (2022). New insights into Hippo/YAP signaling in fibrotic diseases. Cells 11 (13), 2065. 10.3390/cells11132065 35805148 PMC9265296

[B29] Miyamoto S. (2019). Autophagy and cardiac aging. Cell Death Differ. 26 (4), 653–664. 10.1038/s41418-019-0286-9 30692640 PMC6460392

[B30] Moradi N. Fadaei R. Emamgholipour S. Kazemian E. Panahi G. Vahedi S. (2018). Association of circulating CTRP9 with soluble adhesion molecules and inflammatory markers in patients with type 2 diabetes mellitus and coronary artery disease. PLoS One 13 (1), e0192159. 10.1371/journal.pone.0192159 29381773 PMC5790264

[B31] Pesce M. Duda G. N. Forte G. Girao H. Raya A. Roca-Cusachs P. (2023). Cardiac fibroblasts and mechanosensation in heart development, health and disease. Nat. Rev. Cardiol. 20 (5), 309–324. 10.1038/s41569-022-00799-2 36376437

[B32] Qiao S. Hong L. Zhu Y. Zha J. Wang A. Qiu J. (2022). RIPK1-RIPK3 mediates myocardial fibrosis in type 2 diabetes mellitus by impairing autophagic flux of cardiac fibroblasts. Cell Death Dis. 13 (2), 147. 10.1038/s41419-022-04587-1 35165268 PMC8844355

[B33] Shen G. Y. Shin J. H. Song Y. S. Joo H. W. Park I. H. Seong J. H. (2021). Role of autophagy in granulocyte-colony stimulating factor induced anti-apoptotic effects in diabetic cardiomyopathy. Diabetes Metab. J. 45 (4), 594–605. 10.4093/dmj.2020.0049 33631916 PMC8369213

[B34] Stancil I. T. Michalski J. E. Davis-Hall D. Chu H. W. Park J. A. Magin C. M. (2021). Pulmonary fibrosis distal airway epithelia are dynamically and structurally dysfunctional. Nat. Commun. 12 (1), 4566. 10.1038/s41467-021-24853-8 34315881 PMC8316442

[B35] Tallquist M. D. (2020). Cardiac fibroblast diversity. Annu. Rev. Physiol. 82, 63–78. 10.1146/annurev-physiol-021119-034527 32040933 PMC10939057

[B36] Wang H. Wang L. Hu F. Wang P. Xie Y. Li F. (2022). Neuregulin-4 attenuates diabetic cardiomyopathy by regulating autophagy via the AMPK/mTOR signalling pathway. Cardiovasc Diabetol. 21 (1), 205. 10.1186/s12933-022-01643-0 36221104 PMC9554973

[B37] Weng L. Ye J. Yang F. Jia S. Leng M. Jia B. (2023). TGF-β1/SMAD3 regulates programmed cell death 5 that suppresses cardiac fibrosis post-myocardial infarction by inhibiting HDAC3. Circ. Res. 133 (3), 237–251. 10.1161/circresaha.123.322596 37345556

[B38] Wu Z. Liu C. Yin S. Ma J. Sun R. Cao G. (2024). P75NTR regulates autophagy through the YAP-mTOR pathway to increase the proliferation of interfollicular epidermal cells and promote wound healing in diabetic mice. Biochim. Biophys. Acta Mol. Basis Dis. 1870 (3), 167012. 10.1016/j.bbadis.2023.167012 38176461

[B39] Xiang D. Zou J. Zhu X. Chen X. Luo J. Kong L. (2020). Physalin D attenuates hepatic stellate cell activation and liver fibrosis by blocking TGF-β/Smad and YAP signaling. Phytomedicine 78, 153294. 10.1016/j.phymed.2020.153294 32771890

[B40] Xue F. Cheng J. Liu Y. Cheng C. Zhang M. Sui W. (2022). Cardiomyocyte-specific knockout of ADAM17 ameliorates left ventricular remodeling and function in diabetic cardiomyopathy of mice. Signal Transduct. Target Ther. 7 (1), 259. 10.1038/s41392-022-01054-3 35909160 PMC9339545

[B41] Zang H. Wu W. Qi L. Tan W. Nagarkatti P. Nagarkatti M. (2020). Autophagy inhibition enables Nrf2 to exaggerate the progression of diabetic cardiomyopathy in mice. Diabetes 69 (12), 2720–2734. 10.2337/db19-1176 32948607 PMC7679777

[B42] Zhang J. Lyu Z. Li B. You Z. Cui N. Li Y. (2023a). P4HA2 induces hepatic ductular reaction and biliary fibrosis in chronic cholestatic liver diseases. Hepatology 78 (1), 10–25. 10.1097/hep.0000000000000317 36799463

[B43] Zhang L. Zhang H. Xie X. Tie R. Shang X. Zhao Q. (2023b). Empagliflozin ameliorates diabetic cardiomyopathy via regulated branched-chain amino acid metabolism and mTOR/p-ULK1 signaling pathway-mediated autophagy. Diabetol. Metab. Syndr. 15 (1), 93. 10.1186/s13098-023-01061-6 37149696 PMC10163822

[B44] Zhang Q. Wang L. Wang S. Cheng H. Xu L. Pei G. (2022a). Signaling pathways and targeted therapy for myocardial infarction. Signal Transduct. Target Ther. 7 (1), 78. 10.1038/s41392-022-00925-z 35273164 PMC8913803

[B45] Zhang T. He X. Caldwell L. Goru S. K. Ulloa Severino L. Tolosa M. F. (2022b). NUAK1 promotes organ fibrosis via YAP and TGF-β/SMAD signaling. Sci. Transl. Med. 14 (637), eaaz4028. 10.1126/scitranslmed.aaz4028 35320001

[B46] Zhao D. Feng P. Sun Y. Qin Z. Zhang Z. Tan Y. (2018). Cardiac-derived CTRP9 protects against myocardial ischemia/reperfusion injury via calreticulin-dependent inhibition of apoptosis. Cell Death Dis. 9 (7), 723. 10.1038/s41419-018-0726-3 29925877 PMC6010444

