# Supplementary figures and images for: Knockout of C1q/tumor necrosis factor-related protein-9 aggravates cardiac fibrosis in diabetic mice by regulating YAP-mediated autophagy
Source: Front Pharmacol. 2024 Jul 8;15:1407883. doi: 10.3389/fphar.2024.1407883 (PMC11260687; doi:10.3389/fphar.2024.1407883)

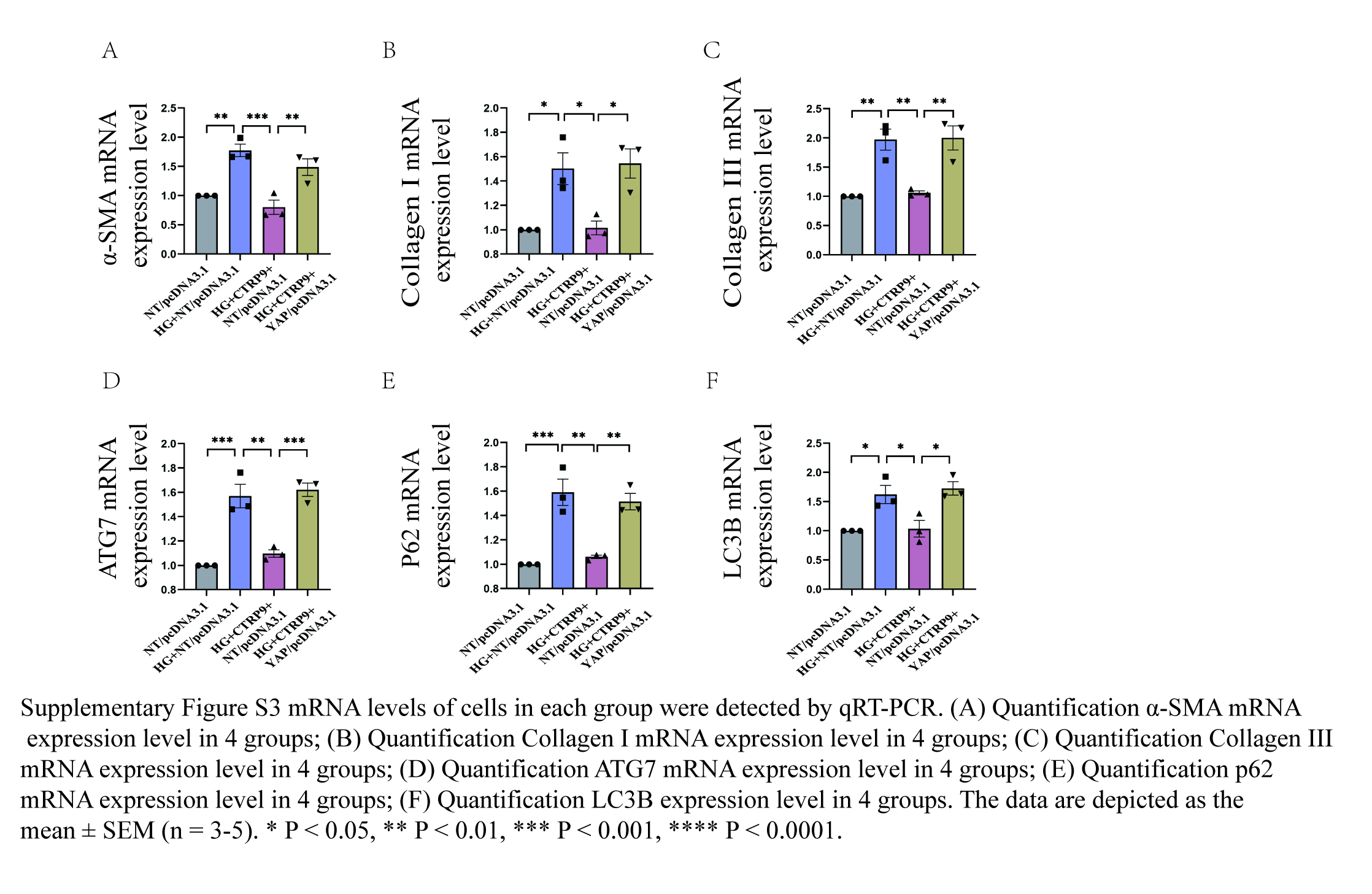

Supplement: Supplementary file 1 [file Image3.tif]

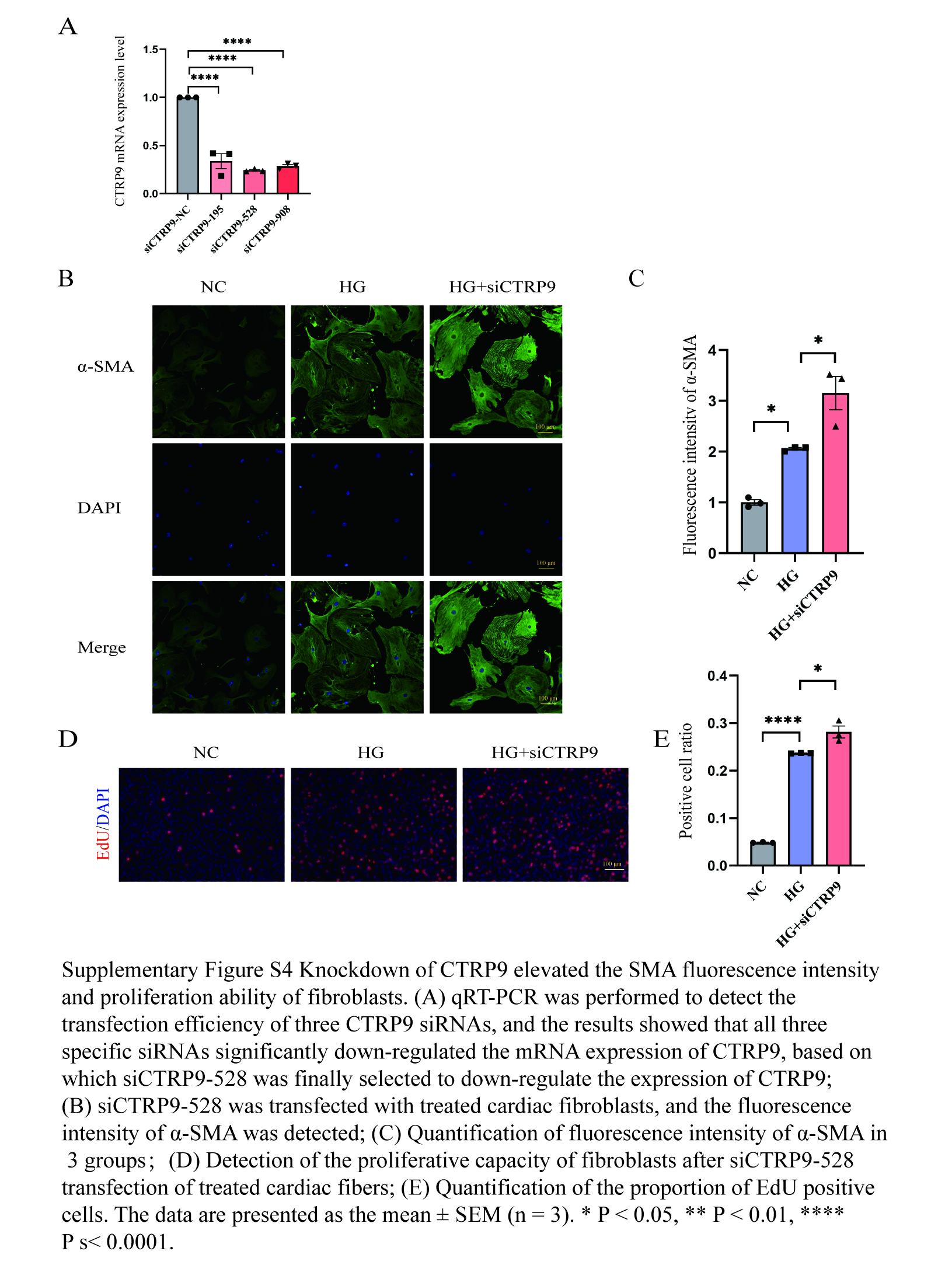

Supplement: Supplementary file 2 [file Image4.tif]

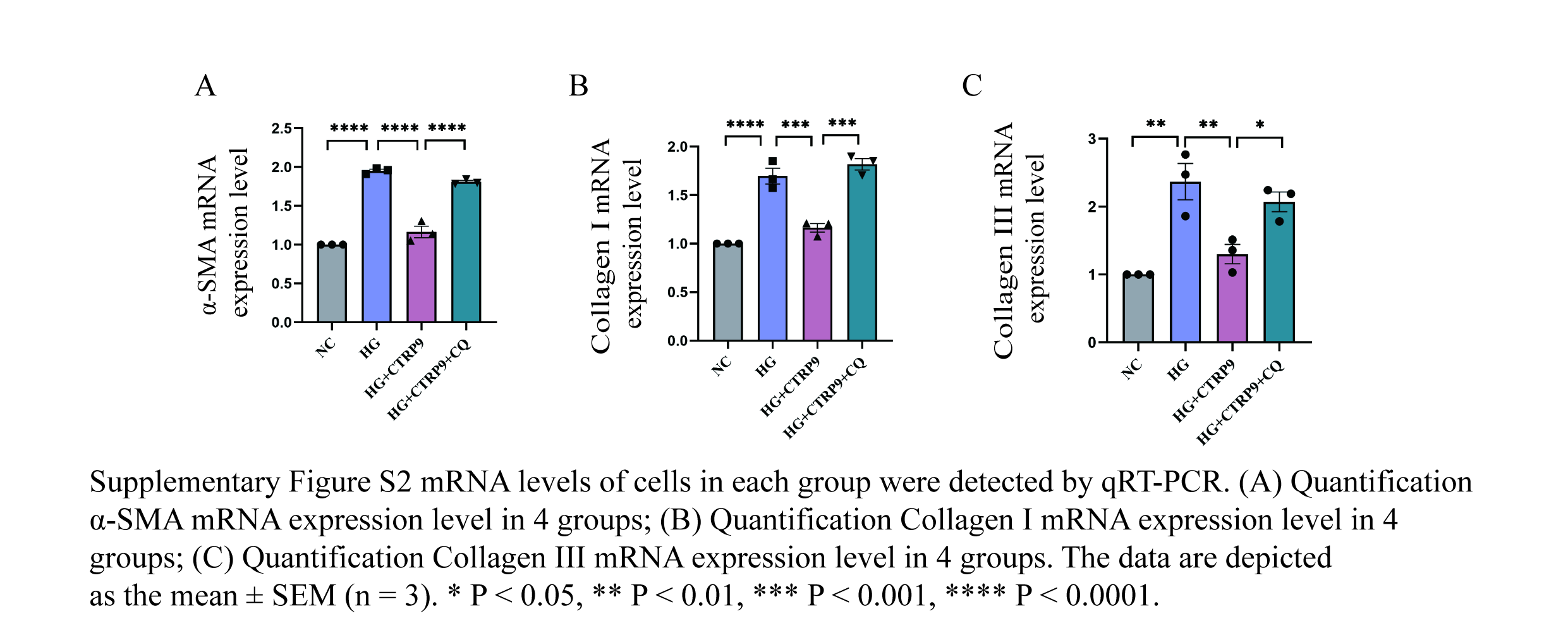

Supplement: Supplementary file 3 [file Image2.tif]

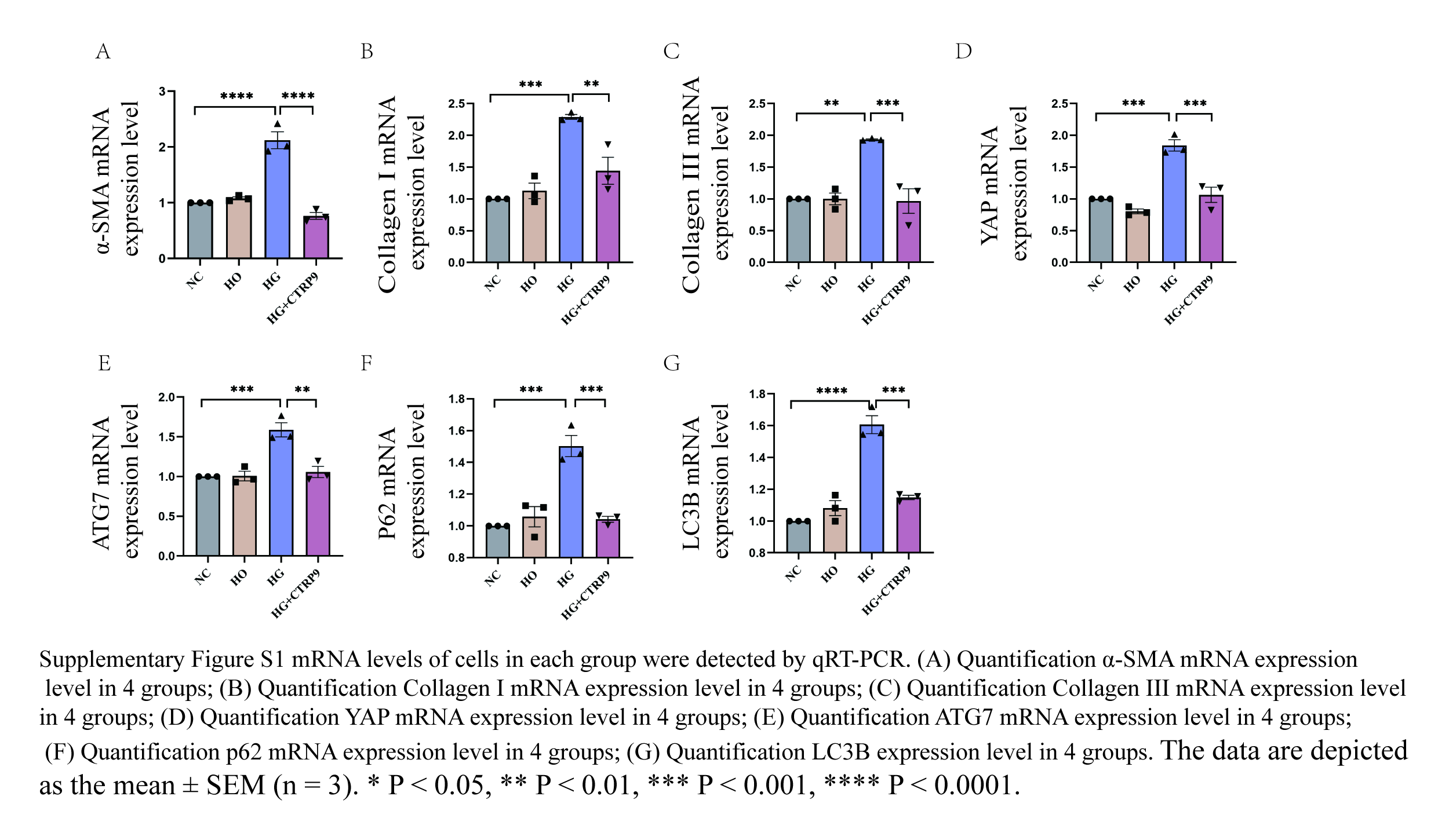

Supplement: Supplementary file 4 [file Image1.tif]

Supplementary material 1: Vimentin 20X

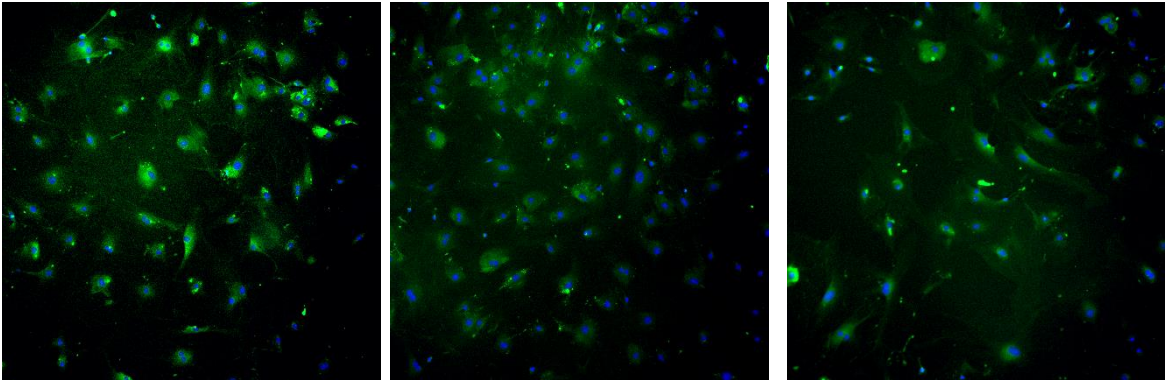

Supplementary material 2: Knockout mouse information

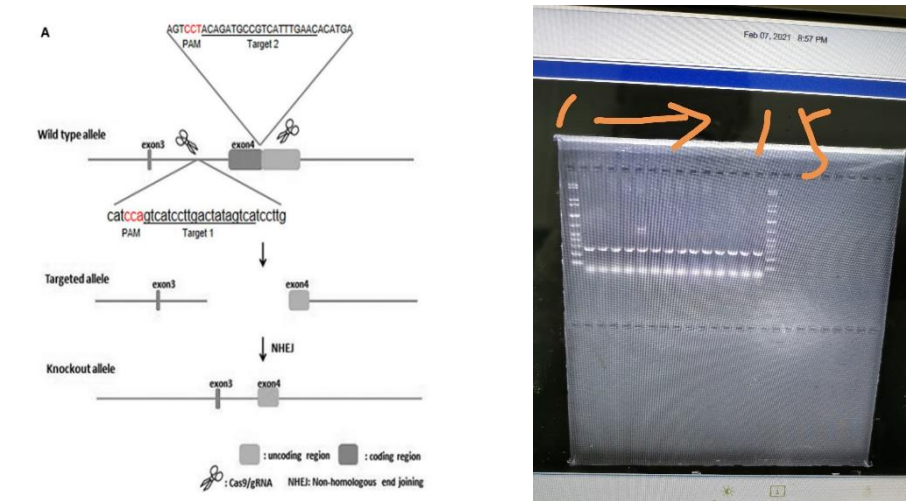

Supplement: Supplementary file 5 [file DataSheet1.pdf]

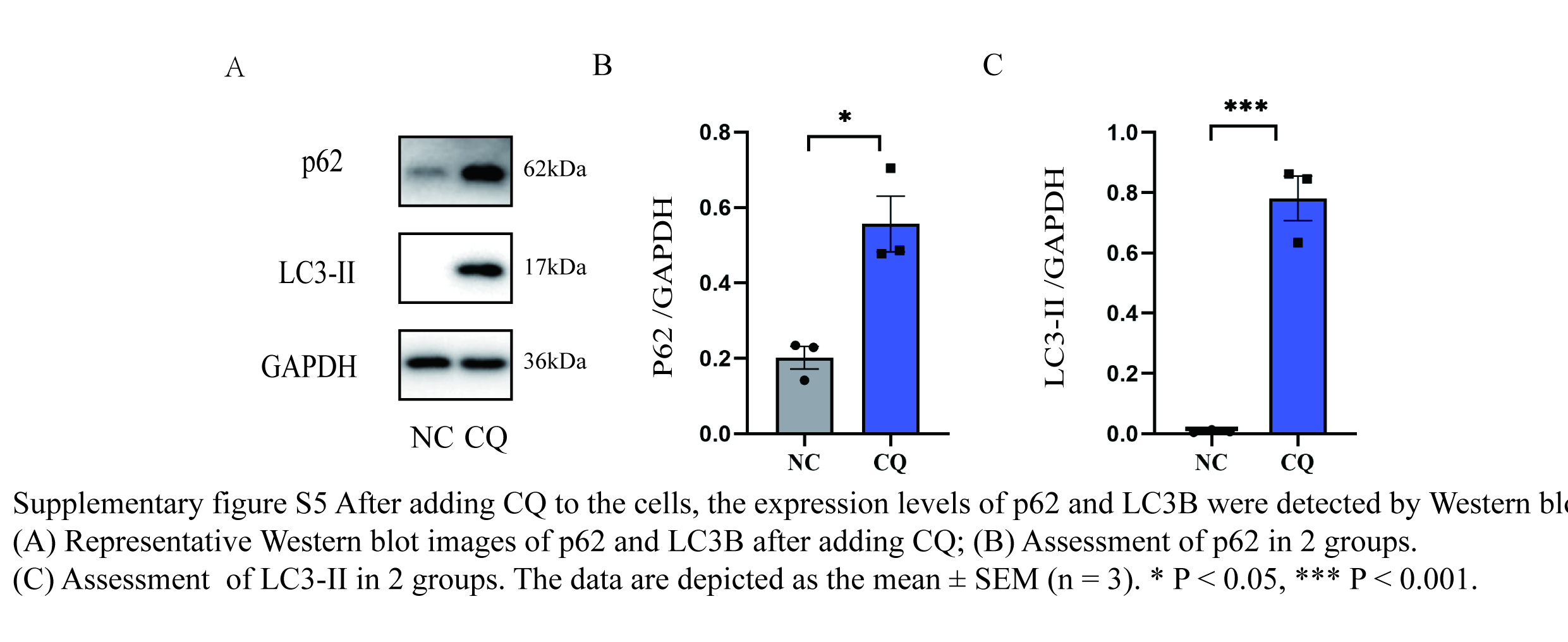

Supplement: Supplementary file 7 [file Image5.tif]
