# Supplementary material for: Knockout of C1q/tumor necrosis factor-related protein-9 aggravates cardiac fibrosis in diabetic mice by regulating YAP-mediated autophagy
Source: Front Pharmacol. 2024 Jul 8;15:1407883. doi: 10.3389/fphar.2024.1407883 (PMC11260687; doi:10.3389/fphar.2024.1407883)
Supplement: Supplementary file 6 [file DataSheet2.docx]

Link to raw data:

<https://www.jianguoyun.com/p/DfH0xMQQvpXWDBiLo9QFIAA>
